# Biomaterials and materiobiology as the continuous powerhouse of biomedical innovation for our aging society

**DOI:** 10.1016/j.fmre.2025.05.008

**Published:** 2025-05-25

**Authors:** Yuanyuan Su, Shuo Li, Guangjun Nie, Ruifang Zhao, Guanghui Ma, Yuliang Zhao, Puyue Wang, Changsheng Liu

**Affiliations:** a Department of Life Sciences, National Natural Science Foundation of China, Beijing 100085, China; b National Center for Nanoscience and Technology, Beijing 100190, China; c State Key Laboratory of Biochemical Engineering, Institute of Process Engineering, Chinese Academy of Sciences, Beijing 100190, China; d East China University of Science and Technology, Shanghai 200237, China

Biomaterials and materiobiology are revolutionizing the approaches to solving aging-related medical challenges. With our society facing increasing aging-related diseases such as cancer, osteoporosis, Alzheimer's disease, Parkinson's disease, diabetes, and cardiovascular diseases, interdisciplinary efforts are crucial to connect material design, biological insights, and clinical unmet needs. This special issue highlights advancements that orchestrate dynamic reciprocity between biomaterials and biomedical needs. By advancing biomaterials design and precision disease targeting, improved diagnostic and therapeutic agents, even with regenerative capabilities, form the foundation to address aging-related diseases [[1], [2], [3], [4], [5], [6], [7]]. By uniting diverse expertise, we aim to catalyze innovations that restore normal tissue function, modulate pathological microenvironments, and deliver targeted therapies, ultimately improving quality of life, especially for aging populations.

These approaches focus on delivering therapies with tissue- or cell-level precision. For example, hyaluronic acid-functionalized metal-drug self-delivery systems target the overexpressed CD44 receptors in hepatocellular carcinoma [1]. These nanostructures accumulated selectively in the cancerous tissues, releasing iron ions to potentiate synergistic chemo/chemodynamic therapy against hepatocellular carcinoma and inhibiting CXCL12/CXCR4 signaling to alleviate the immunosuppressive tumor microenvironment. The targeted mechanism confines drug activity to the tumor region while sparing healthy tissues [2], older patients whose diminished metabolic capacity heightens susceptibility to off-target toxicity. This approach is equally crucial for addressing comorbidities. For example, bone-targeted liposomes leverage alendronate's affinity for hydroxyapatite to deliver senolytic agents to bone, eliminating senescent cells and alleviating therapy-induced bone loss in cancer survivors [3]. These targeted systems illustrate how biomaterials can reduce systemic toxicity and enhance therapeutic efficacy, which is crucial for older patients with polypharmacy and comorbidities.

Targeted delivery can solve only a portion of the clinical challenge. Another equally important aspect is real-time monitoring of disease progression and therapeutic response, particularly in aging populations, where multiple pathologies often coexist and evolve unpredictably. Magnetic nanoparticles leverage their superparamagnetic properties to enable multi-dimensional liver imaging in patients [4]. Beyond detecting hidden tumors, magnetic nanoparticles track the progression of non-alcoholic steatohepatitis toward cirrhosis, offering a comprehensive diagnostic tool for aging-related liver pathologies. Nanozymes serve as theranostic agents, traversing the blood-brain barrier to target neurodegenerative lesions [5]. Once localized, they can sense inflammation and release anti-inflammatory compounds. This capability is amplified by AI tools such as Symbiopersonal Intelligence [6], which analyzes genomic, imaging, and clinical data to predict disease progression and optimize biomaterial designs, translating biological signals into personalized interventions for aging individuals.

Biomaterials should be used simultaneously to treat pathologies and reconstruct structural networks within damaged tissues to restore functionality in aging tissues. DNA framework-assembled hydrogels demonstrate this potential by promoting bone regeneration through programmable mineralization and/or controlled growth factor release [7]. The bone-targeting delivery system shows that eliminating senescent cells stops bone loss and rejuvenates stem cells, enhancing bone regeneration more effectively than traditional drugs [3]. From a broader perspective, real-time sensor feedback, such as inflammation-responsive nanozymes [5] or metabolite-detecting magnetic nanoparticles [4], might jointly enable microenvironment-driven activation of regenerative materials, where therapeutic payloads are dynamically tuned through cross-validating sensor data streams. These advances underscore biomaterials' dual role as disease-modifying agents and architectural scaffolds, offering a synergistic approach to counteract the multifaceted decline of aging tissues.

Collectively, these pieces of work provide valuable insights from biomaterials and materiobiology aspects to anchor innovation for the unmet needs of aging populations. They foresee a future where biomaterials operate as closed-loop systems. These systems will deliver therapies precisely where needed, sense and adapt to dynamic disease states, and ultimately restore tissue integrity. For aging populations, this integration is transformative. With AI tools becoming more personalized, future AI might extend beyond biomaterial optimization. Advanced AI combined with biomaterials could potentially disrupt aging-related pathological cascades by predicting individual senescence signatures, monitoring and neutralizing related biomarkers, or precisely delivering targeted senolytic agents. Yet challenges remain. Scaling these technologies while ensuring safety in older patients with frailty requires deeper collaboration between materials scientists, biologists, and clinicians. As the guest editors, we thank the authors for their groundbreaking contributions, the editorial board for their guidance, and the reviewers for their rigorous comments and timely feedback.

## Declaration of competing interest

The authors declare that they have no conflicts of interest in this work.
